# Inequalities in the provision of rapid HIV testing in primary health care: ecological study, Paraná, 2013-2023

**DOI:** 10.1590/S2237-96222026v35e20250981.en

**Published:** 2026-10-12

**Authors:** Lucas Vinícius de Lima, Gabriel Pavinati, Márcia Moroskoski, Nelly Lopes de Moraes Gil, Iukary Oliveira Takenami, Gabriela Tavares Magnabosco

**Affiliations:** 1 Secretaria Municipal de Saúde, Gerência de Planejamento, Maringá, PR, Brazil; 2 Universidade Estadual de Maringá, Departamento de Enfermagem, Maringá, PR, Brazil; 3 Universidade Federal do Vale do São Francisco, Colegiado de Medicina, Paulo Afonso, BA, Brazil

**Keywords:** HIV, Primary Health Care, Rapid Diagnostic Tests, Spatial Analysis, Time Series Studies, VIH, Atención Primaria de Salud, Pruebas de Diagnóstico Rápido, Análisis Espacial, Estudios de Series Temporales

## Abstract

**Objective:**

To analyze temporal trends, spatial distributions, and factors associated with the provision of rapid HIV testing in primary health care in Paraná, between 2013 and 2023.

**Methods:**

Ecological study using data from the Outpatient Information System (*Sistema de Informações Ambulatoriais*, SIA) on the provision of rapid HIV testing. Testing rates per 10,000 inhabitants were calculated, and temporal trends were analyzed using joinpoint regression. Spatial distribution was performed at the municipal level, and the rates were tested against 17 socioeconomic and programmatic indicators using negative binomial regression.

**Results:**

During the period, 385,960 tests were recorded, of which 89.1% were performed by nurses. The state mean was 30.79 tests per 10,000 inhabitants, with emphasis on the Western macroregion (86.66 per 10,000 inhabitants). Some regions, such as Cornélio Procópio, showed marked growth (annual percentage change [APC] 71.19; 95% confidence interval [95%CI] 55.54; 205.09). Spatial analysis indicated a concentration of municipalities with higher testing rates in the Pato Branco, Francisco Beltrão, and Foz do Iguaçu regions. The illiteracy rate (rate ratio [RR] 0.77; 95%CI 0.63; 0.93), prenatal care coverage (RR 1.20; 95%CI 1.04; 1.37), and detection of acquired syphilis (RR 1.33; 95%CI 1.11; 1.59) were associated with the provision of rapid testing.

**Conclusion:**

Testing showed growth over the study period, though uneven across regions and marked by municipal heterogeneity. Socioeconomic, epidemiological, and programmatic factors influenced performance, indicating the need for targeted policies to reduce inequalities and strengthen primary health care in HIV diagnosis.

## Resumen

**Objetivo:**

Analizar las tendencias temporales, las distribuciones espaciales y los factores asociados a la realización de pruebas rápidas para el VIH en la atención primaria de salud de Paraná, entre 2013 y 2023.

**Métodos:**

Estudio ecológico con datos del Sistema de Información Ambulatoria (*Sistema de Informaciones Ambulatorias*, SIA) sobre la realización de pruebas rápidas para el VIH. Se calcularon las tasas de pruebas por 10.000 habitantes y se analizaron las tendencias temporales mediante regresión segmentada. La distribución espacial se realizó a nivel municipal y sus tasas se contrastaron con 17 indicadores socioeconómicos y programáticos mediante regresión binomial negativa.

**Resultados:**

En el período, se registraron 385.960 pruebas, de las cuales el 89,1% fueron realizadas por enfermeros. La media estatal fue de 30,79 pruebas por 10.000 habitantes, con destaque para la macrorregión Oeste (86,66 por 10.000 habitantes). Algunas regiones, como Cornélio Procópio, mostraron un crecimiento expresivo (variación porcentual anual [VPA] 71,19; intervalo de confianza del 95% [IC95%] 55,54; 205,09). El análisis espacial señaló una concentración de municipios con mayor realización de pruebas en las regiones de Pato Branco, Francisco Beltrão y Foz do Iguaçu. La tasa de analfabetismo (razón de tasas [RT] 0,77; IC95% 0,63; 0,93), la cobertura del control prenatal (RT 1,20; IC95% 1,04; 1,37) y la detección de sífilis adquirida (RT 1,33; IC95% 1,11; 1,59) estuvieron asociadas con la realización de pruebas rápidas.

**Conclusión:**

La realización de pruebas mostró un crecimiento durante el período de estudio, aunque de manera desigual entre regiones y con marcada heterogeneidad a nivel municipal. Factores socioeconómicos, epidemiológicos y programáticos influyeron en el desempeño, lo que indica la necesidad de políticas focalizadas para reducir las desigualdades y fortalecer la atención primaria en el diagnóstico del VIH.

**Aspectos éticos**

This research used public domain anonymized databases.

## Introduction

Since the institutionalization of the National Primary Health Care Policy (*Política Nacional de Atenção Básica*), primary health care (PHC) has been consolidated as a structuring axis of the Brazilian Unified Health System (*Sistema Úncio de Saúde*, SUS)^1^, leading it to assume a key role in the response to communicable diseases. It is recognized as the preferred entry point and the coordinator of care within the SUS. Within the policy framework that established the Family Health Strategy (*Estratégia Saúde da Família*, FHS) as the priority care model, PHC has driven the decentralization and expansion of health services and actions across territories^2^.

Historically, the management of HIV infection was centered in specialized services due to the severity of clinical conditions^3^. However, the evolution of care technologies and the expansion of PHC in the country have enabled a more accessible and locally grounded model^4^. The introduction of rapid testing in PHC can be highlighted and regulated by normative instruments such as Ordinance No. 151, of 14 October 2009^5^, and Ordinance No. 77, of 12 January 2012^6^, which represented milestones in Brazil's response to HIV.

Initially, the provision of rapid tests for HIV and other sexually transmitted infections (STI) was directed to populations in remote areas or in special situations, such as pregnant women and their sexual partners^6^. Over the years, this approach has been expanded and consolidated as a key strategy within PHC^7^. This expansion aimed to strengthen access to early diagnosis of HIV infection, to enable timely interventions, and promote comprehensive care within the SUS.

In the context of the global 95-95-95 target (early diagnosis, antiretroviral therapy, and viral suppression), proposed by the Joint United Nations Programme on HIV/AIDS (UNAIDS)^8^, the decentralization of HIV testing to PHC is essential to achieving the first target: diagnosing 95% of people living with HIV. The reach of PHC, combined with territorial proximity and the capacity to integrate with other levels of care-especially specialized services-enables overcoming access barriers, particularly in regions marked by social inequalities^9^.

Although the decentralization of rapid testing has advanced in Brazil, structural and programmatic challenges continue to compromise equitable access to early HIV diagnosis. Nationally, it is estimated that 1,000,000 people are living with HIV^10^. In 2022, 43,403 individuals were diagnosed^11^; however, disparities in access to HIV diagnosis persist, and the proportion of individuals with a late diagnosis (i.e., at the acquired immunodeficiency syndrome [AIDS] stage) has increased since 2016, reaching 26% in 2023^10^.

In this context, barriers that the SUS must address are evident, with a focus on expanding and strengthening access to early diagnosis. As a result of greater proximity to households and the potential to establish bonds, decentralizing rapid HIV testing to PHC is a highly promising measure to expand access to early diagnosis and timely treatment for the covered population, thereby strengthening practices within the framework of combined prevention.

Studies suggest that stigma, professional insecurity, and heterogeneity in service provision are factors that limit the decentralization of HIV diagnosis to PHC^12^ ^,^ ^13^. Added to this are socioeconomic and programmatic disparities across the Brazilian territory^14^, due to economic, social, and cultural diversity that influences the provision of and access to HIV-related actions and services. This reality requires continuous efforts in professional qualification and effective integration across levels of care.

Despite regulatory advances, the literature on the decentralization of rapid HIV testing to PHC remains limited and heterogeneous, with a concentration of local studies or those restricted to specific populations^3^ ^,^ ^4^ ^,^ ^9^ ^,^ ^12^ ^,^ ^13^. This study, in turn, investigates temporal trends, spatial distributions, and factors associated with the provision of rapid HIV testing in PHC. The combination of these analytical components allows for an understanding of the evolution of testing, its territorial distribution, and the structural and organizational factors that influence it.

## Methods 

Study design

This is an ecological study that employed time series, multiple-group, and analytical cross-sectional analyses, using aggregated data to examine variations over time, distribution across municipalities, and associated factors. This study was based on administrative records, in which all rapid HIV testing procedures recorded in the period were analyzed. The reporting of this study followed the recommendations of the Reporting of Studies Conducted Using Observational Routinely-Collected Health Data (RECORD) checklist.

Setting

In 2022, Paraná had an estimated population of 11,443,208 inhabitants, according to the Brazilian Institute of Geography and Statistics (*Instituto Brasileiro de Geografia e Estatística*, IBGE) population census. In the same year, 2,039 people living with HIV were diagnosed, corresponding to 29.5% of the total notifications in the Southern region; still in that year, 1,830 individuals received an AIDS diagnosis^11^. The health regionalization process in Paraná is structured into four macroregions: East, West, Northwest, and North, with the East subdivided into seven health regions and the other three each comprising five regional health divisions.

The HIV care line, developed by the State Health Secretariat of Paraná, assigns PHC responsibility for prevention and health promotion actions related to HIV and other STI. PHC is also responsible for providing pre- and post-HIV-diagnosis counseling and support, as well as initiating the care cascade at the first appointment for positive serology. In more complex infection cases, affected individuals may be co-managed in partnership with specialized care services.

Data sources and measurement

Data from the Outpatient Information System of the Brazilian Unified Health System (*Sistema de Informações Ambulatoriais do SUS*, SIA/SUS), the Health Information System for Primary Care (*Sistema de Informação em Saúde para a Atenção Básica*, Sisab), the Notifiable Diseases Information System (*Sistema de Informações de Agravos de Notificação*, Sinan), and the IBGE were used, accessed via the Brazilian Unified Health System Information Technology Department (*Departamento de Informação e Informática do SUS*, DATASUS - available at: https://datasus.saude.gov.br/). Data from the Atlas of Human Development (*Atlas do Desenvolvimento Humano*, ADH) (available at: http://www.atlasbrasil.org.br/), accessed on 15 December 2024, were also used.

Population

The study population comprised records of rapid HIV testing procedures from 2013 to 2023, subject to data availability. Procedure code 02.14.01.005-8 ("rapid test for detection of HIV infection") in SIA/SUS was considered, in accordance with Ordinance No. 1,126, of 8 October 2012, of the Health Care Secretariat of the Ministry of Health. Records by municipality of service provision were considered; therefore, the individual's place of residence may differ from the municipality where the procedure was performed.

For the definition of PHC services, the following establishments were considered: "health post," "health center/primary health care unit," "mixed unit," "floating mobile unit," "family health support center," and "Indigenous health care unit." The year of service provision was used for access to SIA/SUS, as production processing may occur at a later time. Based on these selections, no records with missing data were observed.

Variables

The count of rapid HIV tests in the PHC was defined as the dependent variable of the study. Also, from SIA/SUS, information was extracted, stratified by the professional category responsible for testing and by the municipality providing the service. From the municipality level, variables were constructed at the macroregion and health region levels. As independent variables, indicators from the remaining information systems were used, treated as structural contextual variables at the municipal level, according to their respective reference years (Table 1).

**Table 1:** Variables and their respective sources selected from the Health Information System for Primary Care (Sistema de Informação em Saúde para a Atenção Básica), the Notifiable Diseases Information System (Sistema de Informações de Agravos de Notificação), and the Atlas of Human Development (Atlas do Desenvolvimento Humano). Paraná, 2013-2023

Variable	Source (Year)
Socioeconomic/demographic	
Gini Index	ADH (2010)
Percentage of households connected to the piped water supply network	ADH (2010)
Municipal Human Development Index	ADH (2010)
Illiteracy rate in the resident population aged ≥18 years (per 100,000 inhabitants)	ADH (2010)
Expected years of schooling of the population aged 18 years	ADH (2010)
Per capita income (in Brazilian reais)	ADH (2010)
Percentage of people living in poverty (per capita income equal to or below BRL 140.00 per month)	ADH (2010)
Percentage of the resident population aged 15 to 24 years	ADH (2010)
Programmatic/epidemiological	
Percentage of live births with ≥7 prenatal care visits	ADH (2017)
Mortality rate from noncommunicable chronic diseases (per 100,000 inhabitants)	ADH (2017)
Percentage of hospitalizations for conditions sensitive to primary health care	ADH (2017)
AIDS incidence rate (per 100,000 inhabitants)	ADH (2017)
Percentage of supplementary health coverage	ADH (2017)
Percentage of the resident population registered in primary health care	Sisab (2023)
Acquired syphilis detection rate (per 100,000 inhabitants)	Sinan (2022)
Average monthly weighted capitation funding (in Brazilian reais)	Sisab (2023)
Potential coverage of primary health care	Sisab (2023)

Statistical methods

Rapid HIV testing rates were calculated as the ratio of the number of procedures to the resident population in the same location and period, and results were expressed per 10,000 inhabitants. Based on the annual rates for each macroregion/health region, trend analysis was performed using joinpoint regression models. This technique is effective for time series analysis because it allows model adjustment for serial autocorrelation, in accordance with the National Cancer Institute guidelines (https://surveillance.cancer.gov/joinpoint/).

In the time series component, rapid HIV testing rates were treated as the dependent variable in the models, whereas calendar years were treated as the independent variable. The rates were log-transformed to enable comparisons across regions and macroregions. The models were adjusted for the standard errors of these rates and for first-order autocorrelation when identified in the data. The models were estimated using a grid-search approach in the Joinpoint Regression Program, version 5.0.2.

For each final model, annual percentage changes (APC), corresponding to year-to-year changes in rapid HIV testing rates, and their respective 95% confidence intervals (95%CI) were calculated using the empirical quantile method. Positive APC values indicated an increasing trend, whereas negative APC values indicated a decreasing trend. Values whose 95%CI did not include the null value (0.00) were considered statistically significant. Non-significant variations were interpreted as a stationary trend.

In the multiple-group component, annual testing rates were grouped by arithmetic mean (2013-2014 and 2022-2023), representing the beginning and the end of the time series. Based on this approach, two thematic maps were constructed to represent the rates, per 10,000 inhabitants, for each municipality in Paraná. The values for the first biennium were classified using natural breaks, and the same interval parameter was maintained to allow comparison with the second biennium's rates. QGIS software, version 2.36.3, was used for this analysis.

In the cross-sectional ecological analysis, only municipalities with at least one procedure recorded in the SIA/SUS between 2013 and 2023 were included in the modeling of factors associated with the provision of rapid HIV testing in PHC. Given evidence of data overdispersion, negative binomial regression models were used to analyze the association between socioeconomic and programmatic indicators and the number of rapid HIV tests performed in each municipality of Paraná.

In these models, the total count of events during the study period was treated as the dependent variable, socioeconomic and programmatic indicators as the independent variables, and the estimated municipal population as the offset. The independent variables were standardized (*z-scores*) to facilitate comparison of the magnitudes of association and were described using means and standard deviations (SD). They were then subjected to bivariate analyses. Only variables with p-value≤0.200 were considered when constructing parsimonious multivariate models.

Multicollinearity among variables that were marginally significant in the bivariate analysis was assessed using a Spearman correlation matrix and ruled out before multivariate analyses. Model building followed a backward stepwise selection, retaining only independent variables with a p-value≤0.050 in the final model. The measures of association were rate ratios (RR), considered statistically significant when their 95%CI differed from 1.00 (the null value). All statistical analyses were performed using SPSS Statistics software, version 25.1.

## Results 

Between 2013 and 2023, 385,960 rapid HIV testing procedures were recorded in the PHC in Paraná. Analysis of the professionals responsible for testing showed the following distribution: nurses - 89.1%, pharmacists/biochemists - 5.0%, dentists - 3.2%, nursing technicians/assistants - 2.2%, and physicians - 0.5%. The remaining professional categories (physical education, speech-language pathology, physiotherapy, nutrition, social work, and psychology) collectively accounted for 0.0%.

**Figure 1:**
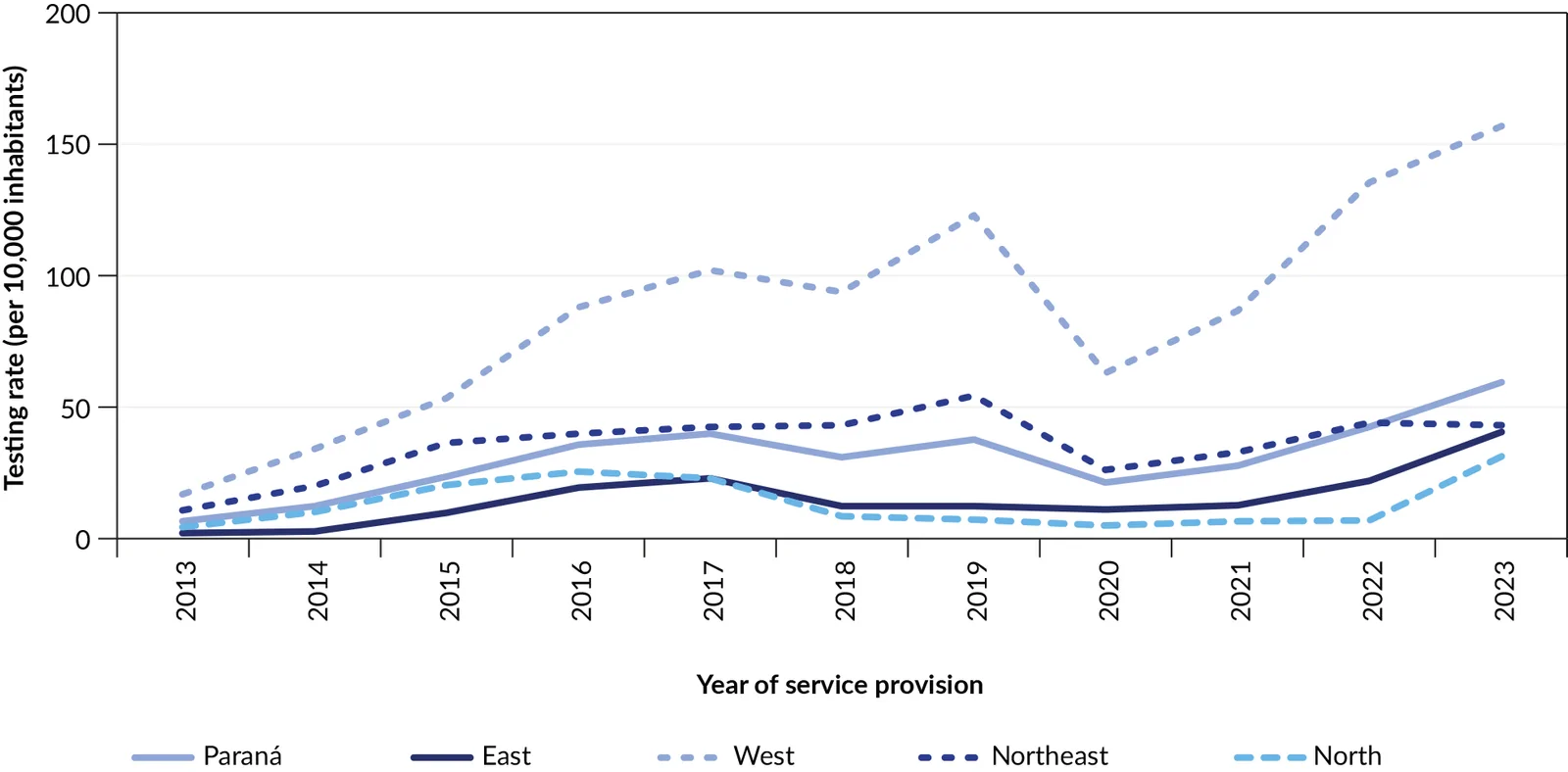
Temporal evolution of rapid HIV testing rates in primary health care by health macroregion. Paraná, 2013-2023 (n=385,960)

Regarding the mean testing rates between 2013 and 2023, Paraná recorded 30.79 HIV tests per 10,000 inhabitants. The West (86.66/10,000 inhabitants) and Northwest (35.86/10,000 inhabitants) macroregions presented mean rates above the state average (Figure 1). Over the study period, the health regions of Pato Branco (7th), Francisco Beltrão (8th), Umuarama (12th), Campo Mourão (11th), Guarapuava (5th), and Toledo (20th) recorded the highest rates. In contrast, Cornélio Procópio (18th), Telêmaco Borba (21st), and Jacarezinho (19th) recorded the lowest rates (Table 2).

Trend analysis revealed that Paraná, as well as the North and Northwest macroregions, maintained a stationary trend in the provision of rapid HIV testing from 2013 to 2023. Increases were observed in the East (APC 16.17; 95%CI 8.11; 56.08) and West (APC 12.85; 95%CI 2.65; 31.87) macroregions. Among the health regions, nine showed an increasing trend, particularly Cornélio Procópio (APC 71.19; 95%CI 55.54; 205.09), whereas Telêmaco Borba and Londrina showed a decreasing trend (Table 2).

In the spatial distribution of HIV testing rates, 327 (81.9%) municipalities recorded no rapid tests during the first biennium (2013-2014). This number decreased in the second biennium analyzed (2022-2023); 275 (68.9%) municipalities had no records of rapid testing in PHC. Overall, there was an improvement in HIV testing between the two biennia, with better performance observed in municipalities in the health regions of Pato Branco (7th), Francisco Beltrão (8th), and Foz do Iguaçu (9th) (Figure 2).

The independent municipal-level variables for municipalities with at least one record in the SIA/SUS are presented in Table 3 (n=173; 43.4%). Among those that were marginally significant in the crude analysis, only three remained in the final model. The adult illiteracy rate was associated with a reduction in HIV testing in PHC (adjusted RR 0.77; 95%CI 0.63; 0.93; p-value 0.007). In contrast, the percentage of live births with ≥7 prenatal care visits (adjusted RR 1.20; 95%CI 1.04; 1.37; p-value 0.008) and the acquired syphilis detection rate (adjusted RR 1.33; 95%CI 1.11; 1.59; p-value 0.001) were associated with higher testing rates (Table 3).

**Table 2:** Annual percentage changes (APC) and 95% confidence intervals (95%CI) of rapid HIV testing rates in primary health care, by macroregion and health region (HR). Paraná, 2013-2023 (n=385,960)

Place of service provision	Testing rates (per 10,000 inhabitants)											Trend	
	2013	2014	2015	2016	2017	2018	2019	2020	2021	2022	2023	APC (95%CI)	Interpretation
East	2.14	2.91	9.72	19.60	23.08	12.46	12.49	11.07	12.89	21.92	40.77	16.17 (8.11; 56.08)	Increasing
1st HR - Paranaguá	0.04	0.04	13.38	29.90	26.75	22.67	18.35	13.67	7.11	2.12	4.79	-13.00 (-31.12; 33.53)	Stationary
2nd HR - Metropolitana	1.18	1.46	3.92	7.11	6.53	8.23	10.61	10.05	11.54	21.30	30.47	29.18 (22.97; 50.82)	Increasing
3rd HR - Ponta Grossa	4.86	12.61	24.92	84.72	97.62	13.79	12.24	0.73	1.39	0.43	1.02	0.94 (-36.34; 69.83)	Stationary
4th HR - Irati	0.06	0.06	0.06	9.45	89.71	33.21	5.32	1.59	0.06	0.06	0.06	-23.54 (-35.75; 516.06)	Stationary
5th HR - Guarapuava	9.51	6.09	37.67	37.92	30.93	36.85	33.05	37.26	34.46	57.01	206.29	29.38 (13.61; 66.02)	Increasing
6th HR - União da Vitória	0.06	0.06	8.47	6.88	26.51	7.03	9.87	19.13	63.76	101.33	120.37	40.10 (30.12; 82.50)	Increasing
21st HR - Telêmaco Borba	0.06	0.06	4.75	0.05	0.11	0.05	0.11	0.05	0.10	0.05	0.10	-44.83 (-68.78; -20.20)	Decreasing
West	16.94	34.24	53.47	88.09	102.00	93.63	122.95	62.91	86.80	135.29	156.97	12.85 (2.65; 31.87)	Increasing
7th HR - Pato Branco	74.85	64.94	149.07	230.14	233.48	287.25	431.01	185.62	247.80	321.76	311.65	9.52 (-1.74; 29.61)	Stationary
8th HR - Francisco Beltrão	25.48	91.27	69.33	127.76	204.35	229.55	246.41	96.05	161.29	295.25	284.84	12.76 (-3.21; 37.94)	Stationary
9th HR - Foz do Iguaçu	7.62	27.95	51.85	57.36	19.77	0.02	0.02	0.02	0.02	23.72	131.41	17.12 (9.92; 45.77)	Increasing
10th HR - Cascavel	0.02	6.91	21.77	43.46	30.79	22.10	43.69	28.24	37.91	68.14	92.46	17.49 (6.75; 35.07)	Increasing
20th HR - Toledo	2.87	5.50	19.72	49.48	103.74	35.29	39.69	62.50	67.46	72.79	54.43	-1.44 (-9.98; 30.90)	Stationary
Northwest	10.97	20.02	36.60	39.90	42.50	43.28	54.49	26.34	33.03	44.21	43.08	5.27 (-2.38; 17.27)	Stationary
11th HR - Campo Mourão	23.30	41.96	63.81	74.73	72.13	72.96	125.31	54.61	73.78	64.65	91.16	6.27 (-2.51; 18.20)	Stationary
12th HR - Umuarama	40.28	60.36	131.19	93.01	99.47	91.00	106.70	51.01	57.13	97.04	60.26	-1.48 (-13.71; 12.23)	Stationary
13th HR - Cianorte	0.53	1.18	4.35	17.48	42.61	45.10	49.68	32.78	27.89	31.37	42.96	0.91 (-7.93; 85.64)	Stationary
14th HR - Paranavaí	0.04	6.59	10.94	11.48	6.03	0.04	0.07	0.04	0.07	33.62	35.20	19.05 (15.19; 54.87)	Increasing
15th HR - Maringa	1.17	4.94	7.73	21.43	23.51	29.58	28.34	14.74	21.40	25.21	21.79	4.68 (-10.02; 33.36)	Stationary
North	4.55	10.34	20.46	25.56	23.02	8.67	7.32	4.96	6.59	6.98	31.20	8.12 (-0.78; 30.95)	Stationary
16th HR - Apucarana	0.60	0.68	2.59	19.48	12.45	4.88	0.03	0.03	0.03	0.03	109.69	35.96 (34.23; 68.15)	Increasing
17th HR - Londrina	1.78	13.86	35.60	36.33	33.78	10.20	8.19	6.47	6.55	5.06	4.61	-19.00 (-34.49; -5.49)	Decreasing
18th HR - Cornélio Procópio	0.04	0.09	0.04	0.09	0.04	0.09	0.04	0.09	0.05	0.09	3.39	71.19 (55.54; 205.09)	Increasing
19th HR - Jacarezinho	0.32	0.63	0.03	0.07	0.03	0.07	0.03	0.07	16.78	27.68	47.94	59.53 (60.79; 116.81)	Increasing
22nd HR - Ivaiporã	49.71	50.27	42.97	65.10	65.51	42.58	51.77	28.45	15.33	8.18	4.93	-4.48 (-26.73; 13.72)	Stationary
Paraná	6.62	12.53	23.68	35.92	39.98	30.94	37.61	21.47	27.85	42.54	59.53	11.64 (-0.54; 35.51)	Stationary

**Figure 2:**
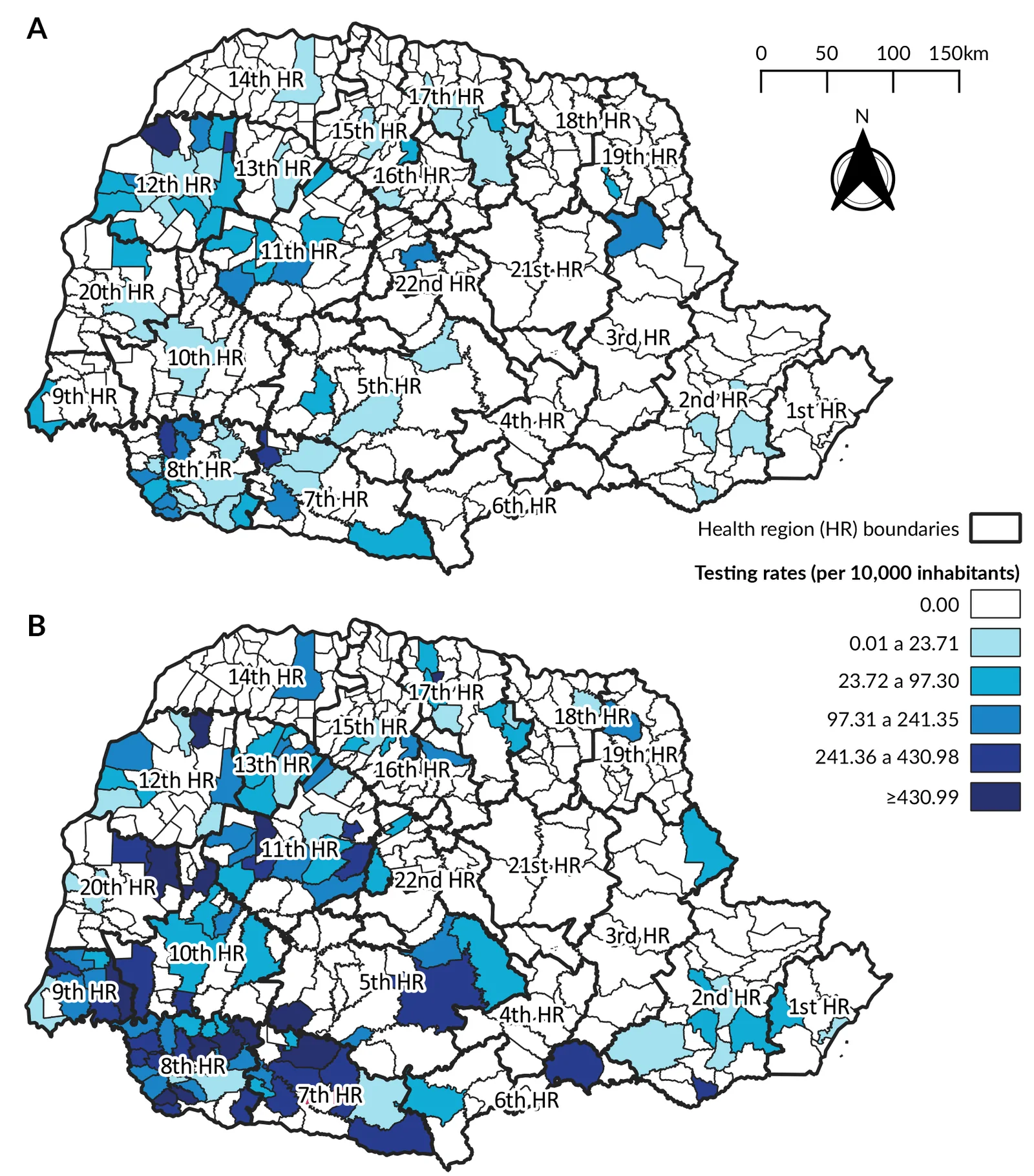
Spatial distribution of rapid HIV testing rates in primary health care in 2013-2014 (A) and 2022-2023 (B) across health regions (HR), by municipality of service provision. Paraná, 2013-2014 and 2022-2023 (n=139,374)

**Table 3:** Means, standard deviations (SD), rate ratios (RR), 95% confidence intervals (95%CI), and p-values for indicators associated with rapid HIV testing in primary health care. Paraná, 2013-2023 (n=385,960)

Indicator	Mean±SD	RR (95%CI)	p-value
Unadjusted analysis			
Gini Index	0.47±0.06	1.12 (0.93; 1.35)	0.204
Percentage of households with access to the piped water supply system	93.73±5.28	1.16 (0.95; 1.43)	0.132
Municipal Human Development Index	0.71±0.04	1.25 (1.06; 1.46)	0.006
Illiteracy rate in the population aged ≥18 years	10.29±4.23	0.76 (0.63; 0.91)	0.003
Percentage of live births with ≥7 prenatal care visits	85.20±7.94	1.12 (0.97; 1.30)	0.095
Mortality rate from noncommunicable chronic diseases	430.04±109.82	0.84 (0.70; 1.02)	0.092
AIDS incidence rate	26.91±198.83	0.95 (0.72; 1.25)	0.734
Percentage of hospitalizations for conditions sensitive to primary health care	22.29±8.60	1.00 (0.83; 1.20)	0.989
Percentage of supplementary health coverage	10.87±9.80	1.18 (1.00; 1.40)	0.040
Expected years of schooling (in years)	10.50±0.86	1.00 (0.84; 1.20)	0.919
Per capita income (in Brazilian reais)	648.59±168.47	1.23 (1.04; 1.45)	0.012
Percentage of the resident population registered in primary health care	100.70±25.22	0.97 (0.82; 1.14)	0.741
Percentage of people living in poverty	9.66±6.29	0.89 (0.74; 1.08)	0.262
Acquired syphilis detection rate	79.38±70.80	1.40 (1.16; 1.69)	<0.001
Percentage of the resident population aged 15-24 years	16.77±0.94	0.90 (0.65; 1.23)	0.514
Average monthly weighted capitation funding (in Brazilian reais)	184,242.25±428,937.80	1.06 (0.91; 1.25)	0.411
Potential coverage of primary health care	96.39±8.23	1.14 (0.93; 1.41)	0.196
Adjusted analysis			
Illiteracy rate in the population aged ≥18 years	10.29±4.23	0.77 (0.63; 0.93)	0.007
Percentage of live births with ≥7 prenatal care visits	85.20±7.94	1.20 (1.04; 1.37)	0.008
Acquired syphilis detection rate	79.38±70.80	1.33 (1.11; 1.59)	0.001

## Discussion 

This study demonstrated advances in the provision of rapid HIV testing in PHC in Paraná, while also highlighting regional inequalities and contextual factors associated with these practices. Overall, significant increases were observed in some regions, although the statewide trend remained stationary. Spatial analysis indicated higher testing rates in border areas, and variables such as lower illiteracy rates, greater prenatal care coverage, and higher acquired syphilis detection rates were associated with HIV testing in PHC.

The West and Northwest macroregions stood out, with rates above the state average. The spatial distribution revealed a greater concentration of tests in border regions, such as Foz do Iguaçu and Pato Branco, located near Paraguay, Argentina, and the Brazilian state of Santa Catarina. Paraná has 139 municipalities sharing borders with South American countries^15^. These territories, characterized by intense population mobility and migratory flows, tend to require more extensive public health surveillance activities.

The use of Brazilian health services by immigrant populations, as a compensatory strategy for disparities in access to health services in their countries of origin, may explain the higher HIV testing rates observed in these regions^16^. Studies have suggested that differences in the organization of health systems across border countries may hinder the implementation of effective public health policies, particularly in controlling communicable diseases through epidemiological surveillance^17^ ^,^ ^18^.

The role of PHC in Paraná's border areas may be considered strategic, as it contributes to the early identification of STI and, consequently, to the interruption of transmission chains. Conversely, a study conducted along the China-Vietnam border reported low HIV testing rates in the region, despite a high prevalence of infection^19^. This finding reinforces the need for more in-depth studies on the role of binational or interstate policies in disease control and PHC coverage.

Low testing rates may reflect structural weaknesses in PHC and difficulties in achieving territorial coverage. The concentration of testing activities among nurses, together with excessive workload demands, may hypothetically limit the expansion of HIV diagnosis in PHC. The limited participation of other health professionals may be related to multiple factors. In addition to potential gaps in training and professional qualification^20^ ^,^ ^21^, organizational factors such as task allocation, overloaded clinical schedules, and the absence of institutional workflows may also restrict the expansion of testing.

Despite the statewide trend remaining stationary, some regions showed marked increases in testing. This result may be attributed to local efforts to improve access to diagnosis and/or the implementation of awareness-raising or community mobilization strategies. These findings suggest that current recommendations for decentralizing HIV care to PHC^22^ ^,^ ^23^ have been implemented in certain localities, resulting in expanded access to early diagnosis.

The stationary trend observed in other regions may be explained by saturation of PHC services or by the absence of local policies to promote testing in areas with lower adherence to national recommendations. It is important to note that limitations in the practice of rapid testing, professional insecurity, and insufficient continuing education have previously been reported as barriers to decentralizing testing^24^. Interventions targeting these challenges may be more effective in promoting equitable access to diagnosis.

The association between higher illiteracy rates and reduced HIV testing highlights the importance of intersectoral programs integrating education and health. Populations with greater access to information tend to make greater use of health services and are more likely to adopt preventive practices^25^. This finding reinforces the importance of strategies to overcome behavioral and social barriers to testing. It also points to potential inequalities in the provision of testing to more vulnerable populations in Paraná.

The percentage of live births with ≥7 prenatal care visits was, in turn, positively associated with HIV testing. This suggests that PHC units with greater problem-solving capacity, as evidenced here by maternal and child health care, perform better at prevention and surveillance. A previous study found that municipalities with greater FHS coverage demonstrated greater resilience in the face of COVID-19-related obstacles^26^, reinforcing the importance of expanding this model.

Higher acquired syphilis detection rates were correlated with greater HIV testing, indicating that territories with active epidemiological surveillance in sexual health achieve better outcomes in prevention and diagnosis. In routine practice, HIV and syphilis testing are often performed concurrently, which may contribute to higher detection rates of both conditions. This finding reinforces the importance of integrating STI control activities into routine PHC practice and highlights that rapid HIV testing is concentrated in municipalities with better PHC performance^27^.

The qualification of FHS teams is also of paramount importance for expanding HIV testing activities and strengthening access to early diagnosis. Consequently, such actions may strengthen the national response toward the sustained achievement of the 95-95-95 targets, particularly the first target. Recently, Brazil achieved two of these targets: 96% of people living with HIV had been diagnosed, of whom 82% were receiving antiretroviral therapy and, among these, 95% had achieved viral suppression, according to UNAIDS estimates for 2023^28^.

The decentralization of rapid HIV testing to PHC may be challenging in universal health systems. In Canada, a strategy involving the distribution of HIV self-tests through online services proved effective in expanding access to early diagnosis^29^. In South Africa, the door-to-door delivery of HIV self-testing by community health workers expanded diagnosis among vulnerable populations^30^. These actions may be adapted to Brazil to reduce inequalities in access, as observed in this study.

The main limitation of this study is its reliance on secondary data, which are subject to underreporting and inconsistencies stemming from data entry and interoperability issues between municipal and federal systems. Such weaknesses may compromise the completeness and representativeness of the information, particularly in municipalities with limited technical and operational capacity. Although there is a specific code in the SIA/SUS for testing in pregnant women and their sexual partners, it is not possible to completely rule out the misclassification of these records as belonging to the general population.

The high proportion of municipalities with no testing records, although it has declined across the biennia analyzed, may also reflect structural limitations in PHC organization or inconsistencies in reporting. The marked variability in annual rates in regions with few procedures increases the instability of estimates, potentially generating artificial fluctuations in temporal trends. These aspects require caution in interpreting the results and reinforce the need for continuous improvement in the quality of information systems.

The limitation related to temporal mismatch among the variables analyzed must be acknowledged. The lack of strict temporal alignment between the dependent and independent variables may introduce inaccuracies in estimating associations, as it does not capture annual variations or recent changes. Such indicators were used as proxies for relatively stable structural characteristics, a common approach in ecological and evaluation studies. Nevertheless, the results should be interpreted with caution, without allowing causal inferences.

Rapid HIV testing in PHC in Paraná showed significant advances, particularly in the West and Northwest macroregions, where indicators such as lower illiteracy rates, better prenatal care performance, and higher syphilis detection contributed to better outcomes. However, regional inequalities and low HIV testing coverage persist in vulnerable areas. The temporal trend indicated statewide stability, with occasional increases that may be attributable to local initiatives. Regions with decreasing trends, such as Telêmaco Borba and Londrina, require priority attention.

These findings reinforce the need to strengthen structured strategies to expand access to early diagnosis in PHC and to reduce territorial disparities. Future research may explore the quality of care pathways between PHC and specialized services, as well as the influence of individual and community barriers to testing, in addition to the impact of municipal combination prevention policies. Qualitative studies and broader epidemiological analyses may further deepen the understanding of the determinants of these regional differences

## Data Availability

The consolidated database used in this study is publicly available at: https://doi.org/10.17632/8s6hj8zbr8.1.
