# Supplementary material for: Inequalities in the provision of rapid HIV testing in primary health care: ecological study, Paraná, 2013-2023
Source: Epidemiol Serv Saude. 2026 Oct 12;35:e20250981. doi: 10.1590/S2237-96222026v35e20250981.en (PMC13671077; doi:10.1590/S2237-96222026v35e20250981.en)
Supplement: Translation

# Translation: Desigualdades na realização de testes rápidos para HIV na atenção primária à saúde: estudo ecológico, Paraná, 2013-2023

**DOI:** 10.1590/S2237-96222026v35e20250981.pt

**Authors:** Lucas Vinícius de Lima, Gabriel Pavinati, Márcia Moroskoski, Nelly Lopes de Moraes Gil, Iukary Oliveira Takenami, Gabriela Tavares Magnabosco

**Affiliations:** 1 Secretaria Municipal de Saúde, Gerência de Planejamento, Maringá, PR, Brasil; 2 Universidade Estadual de Maringá, Departamento de Enfermagem, Maringá, PR, Brasil; 3 Universidade Federal do Vale do São Francisco, Colegiado de Medicina, Paulo Afonso, BA, Brazil

**Keywords:** HIV, Atenção Primária à Saúde, Testes de Diagnóstico Rápido, Análise Espacial, Estudos de Séries Temporais

## Abstract

**Objetivo:**

Analisar tendências temporais, distribuições espaciais e fatores associados à realização de testes rápidos para HIV na atenção primária à saúde do Paraná, entre 2013 e 2023.

**Métodos:**

Estudo ecológico com dados do Sistema de Informações Ambulatoriais sobre realização de testes rápidos para HIV. Foram calculadas as taxas de testagem por 10 mil habitantes, e analisadas as tendências temporais por regressão segmentada. A distribuição espacial foi efetuada sob nível de municípios, e suas taxas foram testadas a 17 indicadores socioeconômicos e programáticos mediante regressão binominal negativa.

**Resultados:**

No período, registraram-se 385.960 testes, dos quais 89,1% foram realizados por enfermeiros. A média estadual foi de 30,79 testes por 10 mil habitantes, com destaque para a macrorregião Oeste (86,66 por 10 mil habitantes). Algumas regiões, como Cornélio Procópio, mostraram crescimento expressivo (variação percentual anual [VPA] 71,19; intervalo de confiança de 95% [IC95%] 55,54; 205,09). A análise espacial apontou concentração de municípios com maior testagem nas regiões de Pato Branco, Francisco Beltrão e Foz do Iguaçu. A taxa de analfabetismo (razão de taxas [RT] 0,77; IC95% 0,63; 0,93), a cobertura de pré-natal (RT 1,20; IC95% 1,04; 1,37) e a detecção de sífilis adquirida (RT 1,33; IC95% 1,11; 1,59) estiveram associadas à realização de testes rápidos.

**Conclusão:**

A testagem apresentou crescimento no período do estudo, embora de forma desigual entre regiões e com forte heterogeneidade municipal. Fatores socioeconômicos, epidemiológicos e programáticos influenciaram o desempenho, o que indicou a necessidade de políticas focalizadas para reduzir desigualdades e fortalecer a atenção primária no diagnóstico do HIV.

Aspectos éticos

Esta pesquisa utilizou bancos de dados de domínio público e anonimizados.

## Introdução 

Desde a institucionalização da Política Nacional de Atenção Básica, a atenção primária à saúde (APS) tem se consolidado como eixo estruturante do Sistema Único de Saúde (SUS)^1^, o que a fez assumir papel central no enfrentamento de doenças transmissíveis. Ela é reconhecida como a porta de entrada preferencial e a ordenadora do cuidado no SUS. A partir da política, que elencou a Estratégia Saúde da Família (ESF) como modelo prioritário de cuidado, a APS tem induzido a descentralização e a capilarização de serviços e de ações de saúde^2^.

Historicamente, o manejo da infecção pelo HIV se centrou em serviços especializados devido à gravidade dos quadros clínicos^3^. A evolução das tecnologias de cuidado e a expansão da APS no país, contudo, tem viabilizado um modelo mais acessível e proximal^4^. Pode-se citar a introdução da testagem rápida na APS, regulamentada por normativas como a Portaria nº 151, de 14 de outubro de 2009^5^, e a Portaria nº 77, de 12 de janeiro de 2012^6^, o que representou marcos no enfrentamento do HIV no Brasil.

Inicialmente, a oferta de testes rápidos para HIV e outras infecções sexualmente transmissíveis (IST) foi destinada a populações em áreas remotas ou em situações especiais, como as gestantes e as suas parcerias sexuais^6^. Ao longo dos anos, essa abordagem foi ampliada e consolidada como uma estratégia-chave na APS^7^. Essa expansão visou fortalecer o acesso ao diagnóstico precoce da infecção pelo HIV, o que visava permitir intervenções oportunas e promover a integralidade do cuidado no âmbito do SUS.

No contexto da meta global 95-95-95 (diagnóstico precoce, terapia antirretroviral e supressão viral), proposta pelo Joint United Nations Programme on HIV/AIDS (UNAIDS)^8^, a descentralização da testagem para HIV à APS é fundamental para alcançar a primeira meta: diagnosticar 95% das pessoas que vivem com HIV. A capilaridade da APS, somada à proximidade territorial e à capacidade de articulação com outros níveis de atenção, sobretudo os serviços especializados, possibilita superar barreiras de acesso, especialmente em regiões marcadas por desigualdades sociais^9^.

Embora a descentralização da testagem rápida tenha avançado no Brasil, desafios estruturais e programáticos continuam a comprometer o acesso equitativo ao diagnóstico precoce do HIV. Nacionalmente, estima-se que 1 milhão de pessoas vivam com HIV^10^. Somente em 2022 foram diagnosticadas 43.403 pessoas^11^, no entanto, ainda existem disparidades no acesso ao diagnóstico do HIV, uma vez que o percentual de indivíduos com diagnóstico tardio (isto é, em estágio de aids) tem aumentado desde 2016, com alcance de 26% em 2023^10^.

Nesse rol, percebem-se entraves a serem superados pelo SUS, com enfoque na expansão e no fortalecimento do acesso ao diagnóstico precoce. Como decorrência da maior proximidade domiciliar e da possibilidade de estabelecimento de vínculo, a descentralização da testagem rápida do HIV à APS se constitui uma medida de grande potencial na ampliação do acesso pela população adstrita ao diagnóstico precoce e ao tratamento oportuno do HIV, o que fortalece as práticas no âmbito do modelo de prevenção combinada.

Estudos sugerem que estigmatização, insegurança profissional e heterogeneidade na oferta dos serviços são fatores que limitam a descentralização do diagnóstico do HIV à APS^12^ ^,^ ^13^. Somam-se a isso as disparidades socioeconômicas e programáticas no território brasileiro^14^, devido à diversidade econômica, social e cultural que influencia a oferta e o acesso às ações e aos serviços relacionados ao HIV. Essa realidade demanda esforços contínuos de qualificação profissional e de integração efetiva entre os níveis de cuidado.

Apesar dos avanços normativos, a literatura sobre a descentralização da testagem rápida do HIV à APS é limitada e heterogênea, com concentração em estudos locais ou restritos a populações específicas^3^ ^,^ ^4^ ^,^ ^9^ ^,^ ^12^ ^,^ ^13^. Esta pesquisa, por sua vez, investiga as tendências temporais, as distribuições espaciais e os fatores associados à realização de teste rápido para HIV na APS. A combinação desses componentes analíticos permite entender a evolução da testagem, sua distribuição territorial e os fatores estruturais e organizacionais que a influenciam.

## Métodos 

Delineamento

Trata-se de um estudo ecológico que compreendeu análises de séries temporais, de múltiplos grupos e transversal analítica, com utilização de dados agregados, para examinar variações ao longo do tempo, distribuição entre municípios e fatores associados. Este é um estudo baseado em registros administrativos, no qual foram analisados todos os procedimentos de testagem rápida para HIV registrados no período. O relato desta pesquisa seguiu as recomendações do checklist *Reporting of Studies Conducted Using Observational Routinely-Collected Health Data* (RECORD).

Contexto

Em 2022, o Paraná possuía população estimada em 11.443.208 habitantes, conforme censo do Instituto Brasileiro de Geografia e Estatística (IBGE). Nesse mesmo ano, foram diagnosticadas 2.039 pessoas vivendo com HIV, o que correspondeu a 29,5% do total de notificações na região Sul; ainda naquele ano, 1.830 pessoas receberam diagnóstico de aids^11^. O processo de regionalização da saúde no Paraná se estrutura em quatro macrorregiões: Leste, Oeste, Noroeste e Norte, com a Leste subdividida em sete regiões de saúde e as outras três possuindo cinco regionais cada.

A linha de cuidado ao HIV, desenvolvida pela Secretaria de Estado da Saúde do Paraná, atribui à APS a responsabilidade por ações de prevenção e de promoção relacionadas ao HIV e a outras IST. Também compete à APS o acolhimento e o aconselhamento pré- e pós-diagnóstico do HIV, bem como o início da cascata de cuidado mediante a primeira consulta em casos de sorologia positiva. Em situações mais complexas da infecção, as pessoas acometidas podem ser comanejadas em parceria com os serviços de assistência especializada.

Fonte de dados e mensuaração

Foram considerados dados oriundos do Sistema de Informações Ambulatoriais do SUS (SIA/SUS), do Sistema de Informação em Saúde para a Atenção Básica (Sisab), do Sistema de Informações de Agravos de Notificação (Sinan) e do IBGE, acessados via Departamento de Informática do SUS - Datasus (disponível em: https://datasus.saude.gov.br/). Também foram utilizados dados do Atlas do Desenvolvimento Humano (ADH) (disponível em: http://www.atlasbrasil.org.br/), acessado em 15 de dezembro de 2024.

População

A população foi composta pelos registros de procedimentos de teste rápido para HIV de 2013 a 2023, conforme a disponibilidade de dados. Considerou-se o código 02.14.01.005-8 ("teste rápido para detecção de infecção pelo HIV") no SIA/SUS, consoante com a Portaria nº 1.126, de 8 de outubro de 2012, da Secretaria de Atenção à Saúde do Ministério da Saúde. Foram considerados os registros por município de atendimento; portanto, a residência da pessoa pode ser diferente do município onde o procedimento foi realizado.

Para fins de definição dos serviços da APS, foram considerados os seguintes estabelecimentos: "posto de saúde", "centro de saúde/unidade básica de saúde", "unidade mista", "unidade móvel fluvial", "centro de apoio à saúde da família" e "unidade de atenção à saúde indígena". O ano de atendimento foi utilizado para acesso ao SIA/SUS, visto que o processamento da produção pode ocorrer em momento posterior. A partir dessas seleções, não foram observados registros de procedimentos com dados ausentes.

Variáveis

A contagem de testes rápidos para HIV na APS foi modulada para servir como variável dependente da pesquisa. Ainda do SIA/SUS, extraíram-se as informações estratificadas por categoria profissional responsável pelo teste e pelo município de atendimento. A partir do município, foram construídas as variáveis em nível de macrorregião e de região de saúde. Como variáveis independentes, foram utilizados os indicadores oriundos dos demais sistemas de informação, tratados como variáveis contextuais estruturais dos municípios, conforme seus respectivos anos de referência (Tabela 1).

**Tabela 1:** Variáveis e respectivas fontes selecionadas no Sistema de Informação em Saúde para a Atenção Básica (Sisab), no Sistema de Informações de Agravos de Notificação (Sinan) e no Atlas do Desenvolvimento Humano (ADH). Paraná, 2013-2023

Variável	Fonte (Ano)
Socioeconômica/demográfica	
Índice de Gini	ADH (2010)
Percentual de domicílios ligados à rede de abastecimento de água encanada	ADH (2010)
Índice de desenvolvimento humano municipal	ADH (2010)
Taxa de analfabetismo na população residente com idade ≥18 anos (por 100 mil habitantes)	ADH (2010)
Expectativa de anos de estudo da população com 18 anos de vida	ADH (2010)
Renda per capita (em reais)	ADH (2010)
Percentual de pessoas pobres (renda per capita igual ou inferior a 140,00 reais mensais)	ADH (2010)
Percentual da população residente com 15 a 24 anos	ADH (2010)
Programática/epidemiológica	
Percentual de nascidos vivos com ≥7 consultas de pré-natal	ADH (2017)
Taxa de mortalidade por doenças crônicas não transmissíveis (por 100 mil habitantes)	ADH (2017)
Percentual de internações por condições sensíveis à atenção primária	ADH (2017)
Taxa de incidência de aids (por 100 mil habitantes)	ADH (2017)
Percentual de cobertura da saúde suplementar	ADH (2017)
Percentual da população residente cadastrada na atenção primária	Sisab (2023)
Taxa de detecção de sífilis adquirida (por 100 mil habitantes)	Sinan (2022)
Financiamento médio mensal da captação ponderada (em reais)	Sisab (2023)
Cobertura potencial da atenção primária	Sisab (2023)

Métodos estatísticos

As taxas de testagem rápida para HIV foram calculadas como a razão entre o número de procedimentos sobre a população residente no mesmo local e período, e o resultado foi expresso por 10 mil habitantes. Com base nas taxas anuais de cada macrorregião/região, realizou-se análise de tendência por modelos de regressão segmentada. Essa técnica é eficaz em análise de séries temporais, visto que permite o ajuste dos modelos para a autocorrelação serial, conforme as orientações do National Cancer Institute (https://surveillance.cancer.gov/joinpoint/).

No componente de séries temporais, as taxas testagem rápida para HIV foram consideradas como a variável dependente nos modelos, enquanto os anos-calendário do período como variável independente. Realizou-se a logaritmização das taxas para permitir a comparação entre as regiões e as macrorregiões. Os modelos foram ajustados pelos erros-padrão dessas taxas e pela autocorrelação de primeira ordem, quando verificada a partir dos dados. Os modelos foram estimados via pesquisa em grade no Joinpoint Regression Program, versão 5.0.2.

Para cada modelo final, calcularam-se, pelo método quantil-empírico, as variações percentuais anuais (VPA) referentes à mudança ano a ano nos valores das taxas de testagem rápida para HIV e os intervalos de confiança de 95% (IC95%). As VPA positivas indicaram tendência crescente, enquanto as VPA negativas, tendência decrescente. Foram considerados significativos os valores cujos IC95% não incluíssem o valor nulo (0,00). As variações não significativas foram interpretadas como tendência estacionária.

No componente de múltiplos grupos, as taxas anuais de testagem foram agrupadas por média aritmética (2013-2014 e 2022-2023), o que representou o início e o fim da série temporal. A partir disso, foram construídos dois mapas temáticos que representaram as taxas, por 10 mil habitantes, em cada município paranaense. Os valores do primeiro biênio foram organizados por meio de quebras naturais, e o parâmetro intervalar foi mantido para proporcionar a comparação com as taxas do segundo biênio. O software QGIS, versão 2.36.3, foi utilizado para essa análise.

No contexto da análise ecológica transversal, para modelar os fatores associados à realização de teste rápido para HIV na APS, consideraram-se apenas os municípios que tiveram, pelo menos, um procedimento registrado no SIA/SUS no período de 2013 a 2023. Perante evidência de superdispersão dos dados, modelos de regressão com distribuição binominal negativa foram utilizados para a análise de associação entre os indicadores socioeconômicos e programáticos e a contagem de testes rápidos para HIV em cada município paranaense.

Nesses modelos, a contagem total do evento no período foi tratada como a variável dependente, os indicadores socioeconômicos e programáticos como as variáveis independentes, e a estimativa da população municipal como o offset. As variáveis independentes foram padronizadas (*z-score*) com vistas a facilitar a comparação das magnitudes de associação e descritas em função da média e do desvio-padrão (DP). Em seguida, elas foram submetidas a análises bivariadas. Somente aquelas com p-valor≤0,200 foram consideradas para a construção de modelos múltiplos parcimoniosos.

A multicolinearidade entre as variáveis marginalmente significativas na análise bivariada foi avaliada mediante matriz de correlação de Spearman e descartada antes das análises múltiplas. A construção dos modelos seguiu a seleção por eliminação regressiva, com preservação somente das variáveis independentes com p-valor≤0,050 no modelo final. As medidas de associação dos modelos foram as razões de taxas (RT), consideradas significantes quando seus IC95% foram diferentes de 1,00 (valor nulo). Todas as análises estatísticas foram realizadas no software SPSS Statistics, versão 25.1.

## Resultados 

Entre 2013 e 2023, foram registrados 385.960 procedimentos de testagem rápida para HIV na APS do Paraná. Ao analisar o profissional responsável pelo atendimento da testagem, evidenciou-se: enfermeiro(a) - 89,1%, farmacêutico(a)/bioquímico(a) - 5,0%, cirurgião(ã)-dentista - 3,2%, técnico(a)/auxiliar de enfermagem - 2,2%, e médico(a) - 0,5%. As demais categorias profissionais (educação física, fonoaudiologia, fisioterapia, nutrição, assistência social e psicologia) totalizaram, juntas, 0,0%.

**Figura 1:** Evolução temporal das taxas de testagem rápida para HIV na atenção primária à saúde, segundo as macrorregiões de saúde. Paraná, 2013-2023 (n=385.960)

Em relação à média das taxas entre 2013 e 2023, o Paraná registrou 30,79 testes de HIV a cada 10 mil habitantes. As macrorregiões Oeste (86,66/10 mil habitantes) e Noroest (35,86/ 10 mil habitantes) apresentaram médias superiores à estadual (Figura 1). No decorrer das séries, as regiões de Pato Branco (7ª), Francisco Beltrão (8ª), Umuarama (12ª), Campo Mourão (11ª), Guarapuava (5ª) e Toledo (20ª) registraram as maiores taxas. Já Cornélio Procópio (18ª), Telêmaco Borba (21ª) e Jacarezinho (19ª) tiveram as menores (Tabela 2).

A análise de tendência revelou que o Paraná, assim como as macrorregiões Norte e Noroeste, mantiveram estacionariedade na realização de teste rápido para HIV de 2013 a 2023. Aumentos foram observados para as macrorregiões Leste (VPA 16,17; IC95% 8,11; 56,08) e Oeste (VPA 12,85; IC95% 2,65; 31,87). Dentre as regiões de saúde, nove apresentaram tendência crescente, com destaque para Cornélio Procópio (VPA 71,19; IC95% 55,54; 205,09), enquanto Telêmaco Borba e Londrina tiveram tendência decrescente (Tabela 2).

Na distribuição espacial das taxas de testagem para HIV, 327 (81,9%) municípios não registraram testes rápidos no primeiro biênio (2013-2014). Esse valor reduziu no segundo biênio analisado (2022-2023), passando para 275 (68,9%) municípios sem registros de teste rápido na APS. De modo geral, houve melhoria na realização de testagem para HIV na comparação entre os biênios, com melhor desempenho em municípios localizados nas regiões de Pato Branco (7ª), Francisco Beltrão (8ª) e Foz do Iguaçu (9ª) (Figura 2).

**Tabela 2:** Variações percentuais anuais (VPA) e intervalos de confiança de 95% (IC95%) das taxas de testagem rápida para HIV na atenção primária à saúde, segundo as macrorregiões e as regiões de saúde (RS). Paraná, 2013-2023 (n=385.960)

Local de atendimento	Taxas de testagem (por 10 mil habitantes)											Tendência	
	2013	2014	2015	2016	2017	2018	2019	2020	2021	2022	2023	VPA (IC95%)	Interpretação
Leste	2,14	2,91	9,72	19,60	23,08	12,46	12,49	11,07	12,89	21,92	40,77	16,17 (8,11; 56,08)	Crescente
1ª RS - Paranaguá	0,04	0,04	13,38	29,90	26,75	22,67	18,35	13,67	7,11	2,12	4,79	-13,00 (-31,12; 33,53)	Estacionária
2ª RS - Metropolitana	1,18	1,46	3,92	7,11	6,53	8,23	10,61	10,05	11,54	21,30	30,47	29,18 (22,97; 50,82)	Crescente
3ª RS - Ponta Grossa	4,86	12,61	24,92	84,72	97,62	13,79	12,24	0,73	1,39	0,43	1,02	0,94 (-36,34; 69,83)	Estacionária
4ª RS - Irati	0,06	0,06	0,06	9,45	89,71	33,21	5,32	1,59	0,06	0,06	0,06	-23,54 (-35,75; 516,06)	Estacionária
5ª RS - Guarapuava	9,51	6,09	37,67	37,92	30,93	36,85	33,05	37,26	34,46	57,01	206,29	29,38 (13,61; 66,02)	Crescente
6ª RS - União da Vitória	0,06	0,06	8,47	6,88	26,51	7,03	9,87	19,13	63,76	101,33	120,37	40,10 (30,12; 82,50)	Crescente
21ª RS - Telêmaco Borba	0,06	0,06	4,75	0,05	0,11	0,05	0,11	0,05	0,10	0,05	0,10	-44,83 (-68,78; -20,20)	Decrescente
Oeste	16,94	34,24	53,47	88,09	102,00	93,63	122,95	62,91	86,80	135,29	156,97	12,85 (2,65; 31,87)	Crescente
7ª RS - Pato Branco	74,85	64,94	149,07	230,14	233,48	287,25	431,01	185,62	247,80	321,76	311,65	9,52 (-1,74; 29,61)	Estacionária
8ª RS - Francisco Beltrão	25,48	91,27	69,33	127,76	204,35	229,55	246,41	96,05	161,29	295,25	284,84	12,76 (-3,21; 37,94)	Estacionária
9ª RS - Foz do Iguaçu	7,62	27,95	51,85	57,36	19,77	0,02	0,02	0,02	0,02	23,72	131,41	17,12 (9,92; 45,77)	Crescente
10ª RS - Cascavel	0,02	6,91	21,77	43,46	30,79	22,10	43,69	28,24	37,91	68,14	92,46	17,49 (6,75; 35,07)	Crescente
20ª RS - Toledo	2,87	5,50	19,72	49,48	103,74	35,29	39,69	62,50	67,46	72,79	54,43	-1,44 (-9,98; 30,90)	Estacionária
Noroeste	10,97	20,02	36,60	39,90	42,50	43,28	54,49	26,34	33,03	44,21	43,08	5,27 (-2,38; 17,27)	Estacionária
11ª RS - Campo Mourão	23,30	41,96	63,81	74,73	72,13	72,96	125,31	54,61	73,78	64,65	91,16	6,27 (-2,51; 18,20)	Estacionária
12ª RS - Umuarama	40,28	60,36	131,19	93,01	99,47	91,00	106,70	51,01	57,13	97,04	60,26	-1,48 (-13,71; 12,23)	Estacionária
13ª RS - Cianorte	0,53	1,18	4,35	17,48	42,61	45,10	49,68	32,78	27,89	31,37	42,96	0,91 (-7,93; 85,64)	Estacionária
14ª RS - Paranavaí	0,04	6,59	10,94	11,48	6,03	0,04	0,07	0,04	0,07	33,62	35,20	19,05 (15,19; 54,87)	Crescente
15ª RS - Maringá	1,17	4,94	7,73	21,43	23,51	29,58	28,34	14,74	21,40	25,21	21,79	4,68 (-10,02; 33,36)	Estacionária
Norte	4,55	10,34	20,46	25,56	23,02	8,67	7,32	4,96	6,59	6,98	31,20	8,12 (-0,78; 30,95)	Estacionária
16ª RS - Apucarana	0,60	0,68	2,59	19,48	12,45	4,88	0,03	0,03	0,03	0,03	109,69	35,96 (34,23; 68,15)	Crescente
17ª RS - Londrina	1,78	13,86	35,60	36,33	33,78	10,20	8,19	6,47	6,55	5,06	4,61	-19,00 (-34,49; -5,49)	Decrescente
18ª RS - Cornélio Procópio	0,04	0,09	0,04	0,09	0,04	0,09	0,04	0,09	0,05	0,09	3,39	71,19 (55,54; 205,09)	Crescente
19ª RS - Jacarezinho	0,32	0,63	0,03	0,07	0,03	0,07	0,03	0,07	16,78	27,68	47,94	59,53 (60,79; 116,81)	Crescente
22ª RS - Ivaiporã	49,71	50,27	42,97	65,10	65,51	42,58	51,77	28,45	15,33	8,18	4,93	-4,48 (-26,73; 13,72)	Estacionária
Paraná	6,62	12,53	23,68	35,92	39,98	30,94	37,61	21,47	27,85	42,54	59,53	11,64 (-0,54; 35,51)	Estacionária

**Figura 2:** Distribuição espacial das taxas de testagem rápida para HIV na atenção primária à saúde em 2013-2014 (A) e em 2022-2023 (B) nas regiões de saúde (RS), segundo o município de atendimento. Paraná, 2013-2014 e 2022-2023 (n=139.374)

As variáveis independentes dos municípios, com ao menos um registro no SIA/SUS, estão na Tabela 3 (n=173; 43,4%). Dentre aquelas marginalmente significativas em análise bruta, apenas três restaram no modelo final. A taxa de analfabetismo entre adultos foi associada a redução na realização do teste na APS (RT ajustada 0,77; IC95% 0,63; 0,93; p-valor 0,007). Em contrapartida, o percentual de nascidos vivos com ≥7 consultas de pré-natal (RT ajustada 1,20; IC95% 1,04; 1,37; p-valor 0,008) e a taxa de detecção de sífilis adquirida (RT ajustada 1,33; IC95% 1,11; 1,59; p-valor 0,001) receberam associação a uma maior testagem (Tabela 3).

**Tabela 3:** Médias, desvios-padrão (DP), razões de taxas (RT), intervalos de confiança de 95% (IC95%) e p-valores dos indicadores associados à testagem rápida para HIV na atenção primária à saúde. Paraná, 2013-2023 (n=385.960)

Indicador	Média±DP	RT (IC95%)	p-valor
Análise bruta			
Índice de Gini	0,47±0,06	1,12 (0,93; 1,35)	0,204
Percentual de domicílios com abastecimento de água	93,73±5,28	1,16 (0,95; 1,43)	0,132
Índice de desenvolvimento humano municipal	0,71±0,04	1,25 (1,06; 1,46)	0,006
Taxa de analfabetismo na população com idade ≥18 anos	10,29±4,23	0,76 (0,63; 0,91)	0,003
Percentual de nascidos vivos com ≥7 consultas de pré-natal	85,20±7,94	1,12 (0,97; 1,30)	0,095
Taxa de mortalidade por doenças crônicas não transmissíveis	430,04±109,82	0,84 (0,70; 1,02)	0,092
Taxa de incidência de aids	26,91±198,83	0,95 (0,72; 1,25)	0,734
Percentual de internações por condições sensíveis à atenção primária	22,29±8,60	1,00 (0,83; 1,20)	0,989
Percentual de cobertura da saúde suplementar	10,87±9,80	1,18 (1,00; 1,40)	0,040
Expectativa de estudo (em anos)	10,50±0,86	1,00 (0,84; 1,20)	0,919
Renda per capita (em reais)	648,59±168,47	1,23 (1,04; 1,45)	0,012
Percentual da população residente cadastrada na atenção primária	100,70±25,22	0,97 (0,82; 1,14)	0,741
Percentual de pessoas pobres	9,66±6,29	0,89 (0,74; 1,08)	0,262
Taxa de detecção de sífilis adquirida	79,38±70,80	1,40 (1,16; 1,69)	<0,001
Percentual da população residente entre 15-24 anos	16,77±0,94	0,90 (0,65; 1,23)	0,514
Financiamento mensal da captação ponderada (em reais)	184.242,25±428.937,80	1,06 (0,91; 1,25)	0,411
Cobertura potencial da atenção primária	96,39±8,23	1,14 (0,93; 1,41)	0,196
Análise ajustada			
Taxa de analfabetismo na população com idade ≥18 anos	10,29±4,23	0,77 (0,63; 0,93)	0,007
Percentual de nascidos vivos com ≥7 consultas de pré-natal	85,20±7,94	1,20 (1,04; 1,37)	0,008
Taxa de detecção de sífilis adquirida	79,38±70,80	1,33 (1,11; 1,59)	0,001

## Discussão 

Este estudo evidenciou avanços na realização da testagem rápida para HIV na APS do Paraná, ao mesmo tempo em que destacou desigualdades regionais e fatores contextuais associados a essas práticas. De modo geral, houve aumento significativo em algumas regiões, embora a tendência estadual tenha se mantido estacionária. A análise espacial indicou maior testagem em áreas fronteiriças, e variáveis como menor taxa de analfabetismo, maior cobertura de pré-natal e maior detecção de sífilis adquirida foram associadas à testagem na APS.

As macrorregiões Oeste e Noroeste se destacaram com taxas superiores à média estadual. A distribuição espacial revelou maior concentração de testes nas regiões fronteiriças, como Foz do Iguaçu e Pato Branco, próximas ao Paraguai, à Argentina e a Santa Catarina, no Brasil. O Paraná possui 139 municípios com fronteira compartilhada com países sul-americanos^15^. Esses territórios, caracterizados por intensa mobilidade populacional e presença de fluxos migratórios, tendem a exigir mais ações de vigilância em saúde.

A participação da população imigrante nos serviços de saúde brasileiros, como uma estratégia compensatória para as disparidades no acesso aos serviços em seus países de origem, pode justificar a maior testagem para HIV nessas regiões^16^. Estudos sugeriram que as diferenças na organização dos sistemas de saúde nos países fronteiriços podem dificultar a implementação de políticas públicas de saúde eficazes, sobretudo no que tange ao controle de doenças transmissíveis mediante as ações de vigilância epidemiológica^17^ ^,^ ^18^.

A atuação da APS nas áreas de fronteira do Paraná pode ser considerada estratégica, pois contribui para a identificação precoce das IST e, consequentemente, para a interrupção das cadeias de transmissão. Contrariamente, estudo realizado na fronteira entre China e Vietnã apontou para a baixa testagem para HIV na região, ao passo que a prevalência da infecção se mostrou elevada^19^. Isso reforça a necessidade de estudos mais aprofundados sobre a atuação das políticas binacionais ou interestaduais no controle de doenças e na cobertura da APS.

As baixas taxas podem refletir fragilidades estruturais da APS e dificuldades de alcance territorial. A centralização da testagem no enfermeiro e a sobrecarga assistencial podem, hipoteticamente, limitar a ampliação do diagnóstico na APS. A participação limitada de outros profissionais pode estar relacionada a múltiplos fatores. Além de eventuais lacunas na formação e na qualificação^20^ ^,^ ^21^, aspectos organizacionais, como divisão de tarefas, agendas assistenciais sobrecarregadas e ausência de fluxos institucionais, também podem restringir a expansão da testagem.

Apesar da estabilidade na tendência estadual, algumas regiões apresentaram crescimento expressivo na testagem. Esse resultado pode ser atribuído a esforços locais para melhorar o acesso ao diagnóstico e/ou à implementação de estratégias de sensibilização ou mobilização comunitária. Esses dados sugerem que as recomendações vigentes para a descentralização do cuidado ao HIV para a APS^22^ ^,^ ^23^ têm sido acatadas em determinadas localidades, com ampliação no acesso ao diagnóstico precoce.

A estacionariedade observada em outras regiões pode ser explicada por saturação da APS ou por falta de políticas locais para estimular a testagem em áreas com menor adesão às recomendações nacionais. É importante destacar que a limitação da prática do teste rápido, a insegurança dos profissionais e a escassa educação permanente já foram reportadas como limitadoras da descentralização da testagem^24^. Intervenções direcionadas a essas problemáticas podem ser mais eficazes para promover o acesso equitativo ao diagnóstico.

A associação entre o aumento na taxa de analfabetismo e a redução na realização de testes para HIV destaca a importância de programas intersetoriais que integrem educação e saúde. Populações com maior acesso à informação tendem a buscar mais os serviços de saúde e a aderir a práticas preventivas^25^. Esse achado reforça a importância de estratégias que visem superar as barreiras comportamentais e sociais à testagem. Aponta também para as possíveis desigualdades na oferta do teste a populações mais vulneráveis no Paraná.

O percentual de nascidos vivos com ≥7 consultas de pré-natal, por sua vez, esteve associado positivamente com a testagem. Isso sugere que as unidades de APS com maior resolutividade, aqui evidenciada pelo cuidado materno-infantil, têm melhor desempenho em atividades de prevenção e de vigilância. Estudo prévio apontou que os municípios com maior cobertura da ESF demonstraram maior resiliência frente aos obstáculos impostos pela pandemia de covid-19^26^, o que reforça a essencialidade da expansão desse modelo.

A maior detecção de sífilis adquirida se correlacionou com a maior realização de testes para HIV, o que indica que territórios com vigilância epidemiológica ativa em saúde sexual apresentam melhores resultados em prevenção e em diagnóstico. Rotineiramente, a testagem para HIV e para sífilis é concomitante, o que poderia implicar na maior detecção delas. Isso reforça a importância de integrar as ações de controle de IST à rotina da APS e sublinha que a realização de teste rápido para HIV se concentra em municípios com melhor desempenho da APS^27^.

A qualificação das equipes da ESF também se mostra de suma importância para ampliar as ações de testagem para HIV e para fortalecer o acesso ao diagnóstico precoce. Por conseguinte, tais ações podem impulsionar a resposta nacional no alcance sustentado das metas 95-95-95, especificamente o primeiro objetivo. Recentemente, o Brasil já atingiu duas dessas metas: 96% das pessoas foram diagnosticadas, das quais 82% estavam em terapia antirretroviral e, destas, 95% estavam com a carga viral suprimida, conforme as estimativas do UNAIDS de 2023^28^.

A descentralização da testagem rápida para HIV na APS pode ser desafiadora em sistemas universais de saúde. No Canadá, uma estratégia de dispensação de autoteste para HIV por meio de serviços on-line foi eficaz para ampliar o acesso ao diagnóstico precoce^29^. Já na África do Sul, a entrega porta a porta de autoteste para HIV por meio de agentes comunitários expandiu o diagnóstico em populações vulneráveis^30^. Essas ações podem ser adaptadas ao Brasil para reduzir desigualdades no acesso, como observado neste estudo.

A principal limitação desta pesquisa decorre do uso de dados secundários, sujeitos a sub-registros e a inconsistências associadas à alimentação e à interoperabilidade entre sistemas municipais e federais. Tais fragilidades podem comprometer a completude e a representatividade das informações, sobretudo em municípios com baixa capacidade técnico-operacional. Embora exista código específico no SIA/SUS para testagem em gestantes e em suas parcerias sexuais, não é possível descartar totalmente a classificação indevida desses registros como pertencentes à população geral.

A elevada proporção de municípios sem registros de testagem, ainda que reduzida ao longo dos biênios analisados, também pode refletir limitações estruturais na organização da APS ou inconsistências de registro. A expressiva variabilidade das taxas anuais em regiões com pequeno número de procedimentos amplia a instabilidade das estimativas, o que pode gerar oscilações artificiais nas tendências temporais. Esses aspectos exigem cautela na interpretação dos resultados e reforçam a necessidade de aprimoramento contínuo da qualidade dos sistemas de informação.

Deve-se reconhecer a limitação relacionada à disparidade temporal entre as variáveis analisadas. A ausência de compatibilidade temporal estrita da variável dependente com as independentes pode introduzir imprecisões na estimativa das associações, uma vez que não capta variações anuais ou mudanças recentes. Tais indicadores foram utilizados como medidas indiretas de características estruturais relativamente estáveis, abordagem recorrente em estudos ecológicos e de avaliação. Ainda assim, os resultados devem ser interpretados com cautela, sem a permissão de inferências causais.

A testagem rápida para HIV na APS do Paraná apresentou avanços expressivos, especialmente nas macrorregiões Oeste e Noroeste, onde indicadores como menor analfabetismo, melhor desempenho no pré-natal e maior detecção de sífilis contribuíram para melhores resultados. Persistem, contudo, desigualdades regionais e baixa cobertura da testagem para HIV em áreas vulneráveis. A tendência temporal indicou estabilidade estadual, com aumentos pontuais atribuíveis, possivelmente, a iniciativas locais. Regiões com tendência decrescente, como Telêmaco Borba e Londrina, requerem atenção prioritária.

Esses achados reforçaram a necessidade de fortalecer estratégias estruturadas de ampliação do acesso ao diagnóstico precoce na APS e de reduzir disparidades territoriais. Pesquisas futuras podem explorar a qualidade dos fluxos assistenciais entre APS e serviços especializados e a influência de barreiras individuais e comunitárias na busca pelo teste, além do impacto de políticas municipais de prevenção combinada. Estudos qualitativos e análises epidemiológicas mais extensas também podem aprofundar a compreensão dos determinantes dessas diferenças regionais.

## Data Availability

O banco de dados consolidado e utilizado neste estudo está disponível publicamente em: https://doi.org/10.17632/8s6hj8zbr8.1.
